# Exhaled contaminant concentration data in a hospital room influenced by external heat gains

**DOI:** 10.1016/j.dib.2019.103978

**Published:** 2019-05-08

**Authors:** Ines Olmedo, Felix A. Berlanga, Jose Manuel Villafruela, Manuel Ruiz de Adana

**Affiliations:** a Department of Chemical Physics and Applied Thermodynamics, Cordoba University, Spain; b Department of Energy and Fluid Mechanics, University of Valladolid, 47004 Valladolid, Spain

**Keywords:** Intake fraction, Exhaled contaminants distribution, External heat gains

## Abstract

A hospital room is simulated using two breathing thermal manikins representing a health worker (HW) and a patient in repose (PR). The PR exhales R134a simulating small exhaled particles (<5 μm) and the contaminant concentration around and in the inhalation of HW is measured during a period of 2 hours per experiment. The room is climatized to maintain constant air temperature values of 25 °C using two wall mounted grilles and two exhausts. The air change per hour (ACH) used varies from 6 to 12 in order to evaluate its influence on the dispersion of exhaled contaminants. An external heat gain is simulated by a radiant wall (RW) that is active or inactive during the experiments. When RW is active the external heat gain simulated is 39.7 W/m^2^, which corresponds to a solar external radiation typical in many countries worldwide. The contaminant concentration is measured at different locations around HW and in the inhalation tube. The temperature gradient in the room is also measured along a vertical pole. The discussion of the results of the 16 experimental cases can be found in Ref. [1].

**Table:** Specifications Table

Subject area	*Physics*
More specific subject area	*Distribution of contaminants in indoor environments*
Type of data	*Excel files, table, figure*
How data was acquired	Innova 1303, LumaSense Technologies, California Thermocouples type J
Data format	*Raw Analyzed (Excel files)* • Concentration_results.xls • Temperature_results.xls • Inh_transient_results.xls
Experimental factors	*Tests 2: The contaminant concentration in the inhalation of HW is measured during a period of 2 hours, obtaining 204 samples.* *Tests 1: The contaminant concentration is measured in different positions of the room, including the exhaust, over 6 hours.*
Experimental features	*Two thermal breathing manikins representing a health worker (HW) and a patient at rest (PR) in a simulated hospital room. PR exhales contaminants that are measured at different positions around HW. The room is ventilated by two wall mounted grilles using 6 and 12 ACH. The tests are carried out considering a radiant wall (RW) active and inactive in order to simulate the influence of external heat gains on the dispersion of exhaled contaminants.*
Data source location	*Córdoba (Spain)*
Data accessibility	*Data is available with this article*
Related research article	I. Olmedo, F.A. Berlanga, J.M. Villafruela, M. Ruiz De Adana, Experimental variation of the personal exposure in a hospital room influenced by wall heat gains, *Building and Environment*, 154, 2019, 252–262 [1].

**Table:** 

**Value of the data** • The presented data are transient measurements of experimental tests that are valuable in order to understand the transient nature of dispersion of exhaled and inhaled contaminants. • This data may be very useful to validate a numerical model • Modifications to the ventilation conditions may be carried out and compared to the results obtained here.

## 1 Data

The data shared in this article is presented in three excel files. Each file compiles a variable measured and processed. The three excel files are briefly described using tables to describe each nomenclature:

• FILE 1: “**Concentration_results.xls**”, which presents the values of contaminant concentration measured in different positions of the room for the 16 experimental tests carried out. In this file it is possible to find different columns to select the data that want to be obtained. For column E the possibilities to select the data are:
  ○ Test 1: HW bell in P1, HW neck in P1, HW head in P1, O exhaust and N exhaust.
  ○ Test 2. HW inhalation.

In order to avoid the complete amount of data obtained in each case, which correspond to transient measurements over time, the results are presented using the average and the standard deviation of the individual results in each case. The relation between the symbols used for each variable in Ref. [1], the symbols used in the Excel file and the detailed explanation of how raw data is treated is found in Table 1.

• FILE 2: “**Temperature_results.xls**”, which contains the temperature values registered inside the experimental chamber for the experiments developed. Again, and in order to simplify the data obtained, the data is provided as an average of the individual temperature registers obtained in each experiment. The temperature material is provided for the four different thermal situations which corresponds to the cases: RW ON_6, RW OFF_6, RW ON_12 and RW OFF_12. The relation between the symbols used for each variable in Ref. [1], the symbols used in the Excel document and the detailed explanation of how raw data is treated is found in Table 2.

**Table 2:** Nomenclature of “Temperature_results.xls” file.

Excel Symbol	Meaning of the variable
T_i	Average value of temperature for *i* point (°C), $T_{i}$
T_in	Average value of temperature in the supply (°C), $T_{in}$
T_out	Average value of temperature in the exhaust (°C), $T_{out}$
• FILE 3: “**Inh_transient_results.xls**”, which presents the 204 transient samples of the measurements of ci_nh_ and e_inh_ during test 2 for each of the 16 experimental cases. In this excel file can be found the results of the amount of contaminant inhaled by HW for the different experimental situation that are described in Table 3.

**Table 3:** Nomenclature of “Inh_transient_results.xls” file.

Air change per hour in the room	External heat gains condition	HW position	Excel sheet name
6 ACH	RW ON	NM	RW ON_6 NM
		NC	RW ON_6 NC
		OM	RW ON_6 OM
		OC	RW ON_6 OC
	RW OFF	NM	RW OFF_6 NM
		NC	RW OFF_6 NC
		OM	RW OFF_6 OM
		OC	RW OFF_6 OC
12 ACH	RW ON	NM	RW ON_12 NM
		NC	RW ON_12 NC
		OM	RW ON_12 OM
		OC	RW ON_12 OC
	RW OFF	NM	RW OFF_12 NM
		NC	RW OFF_12 NC
		OM	RW OFF_12 OM
		OC	RW OFF_12 OC

**Table 1:** Nomenclature of “concentration_results.xls” file.

Excel Symbol	Meaning of the variable
c_i	Average value of tracer gas concentration gathered for *i* point (ppm), $c_{i}$
S(c_i)	Standard deviation of the tracer gas concentration registers during the test for *i* point
c_max	Maximum contaminant concentration (ppm) registered for each of the measuring points including $c_{inh\_\max}$
c_125%	Average tracer gas concentration of the values that exceed in 25% the mean average concentration ($e_{inh125\%}$)
e	Average tracer gas exposure of HW, ${\bar{e}}_{\mathrm{inh}}$
e_125%	Average exposure of the values that exceed in 25% the mean personal exposure of HW, $e_{inh125\%}$
e_max	Maximum personal exposure, $e_{inh\_\max}$
IF	Average intake fraction
IF_125%	Average intake fraction of the values that exceed in 25% the mean intake fraction of HW, $IF_{125\%}$
IF_max	Maximum intake fraction, $IF_{m\mathrm{ax}}$

## 2 Experimental design, materials and methods

**Fig. 1:**
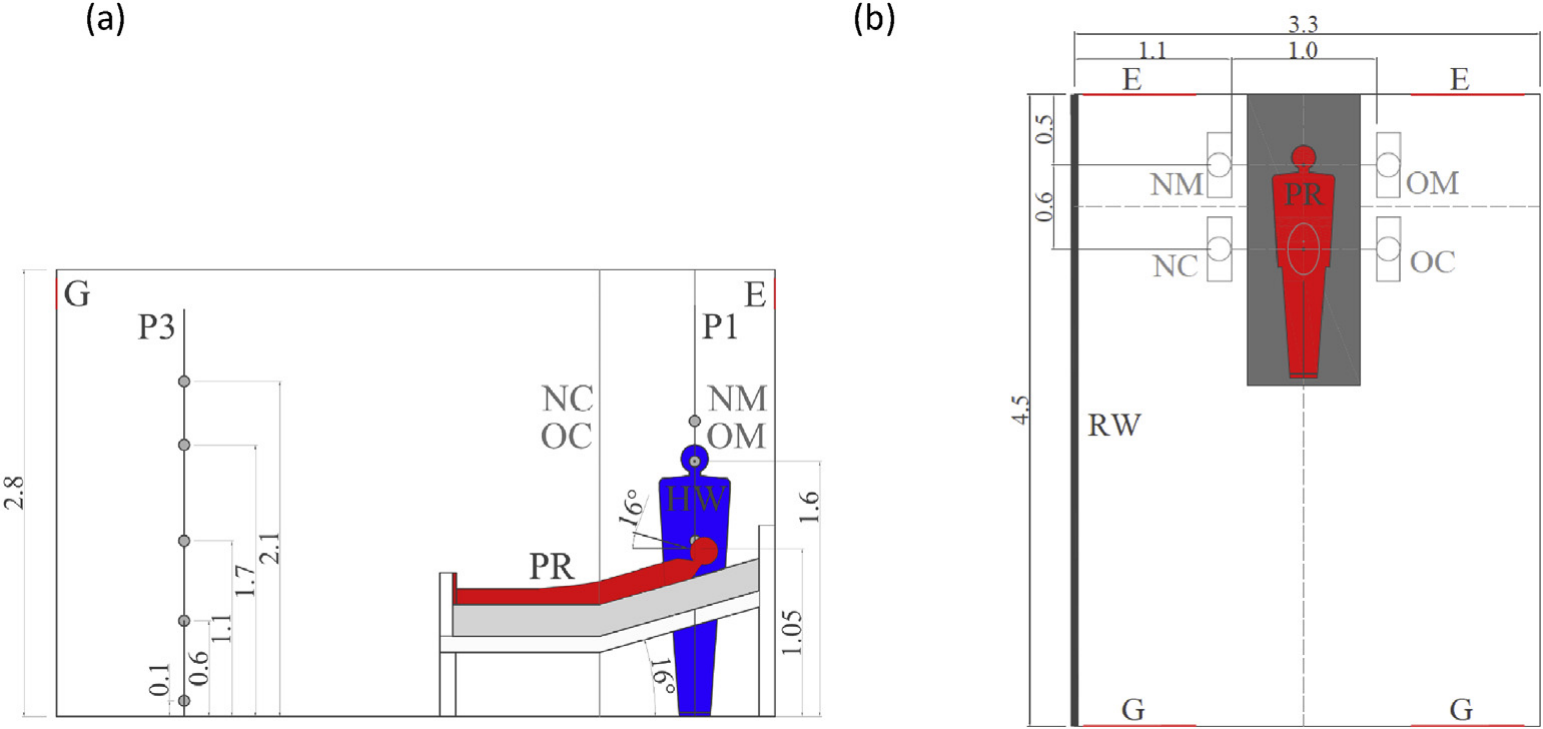
(a) Setup of the test room with the two thermal breathing manikins (PR: source manikin in red (exhalation height of 1.05 m), and HW: target manikin in blue), the radiant wall (RW), the supply grilles (G) and exhausts (E), and the two vertical poles used for measurements: P1 and P3. (b) HW positions: NC, NM, OC and OM. All measurements are in meters.

Two thermal breathing manikins are placed in an experimental hospital room. One manikin represents a patient in repose (PR) and the other manikin a standing health worker (HW). The layout of the experimental room is shown in Fig. 1.

**Fig. 2:**
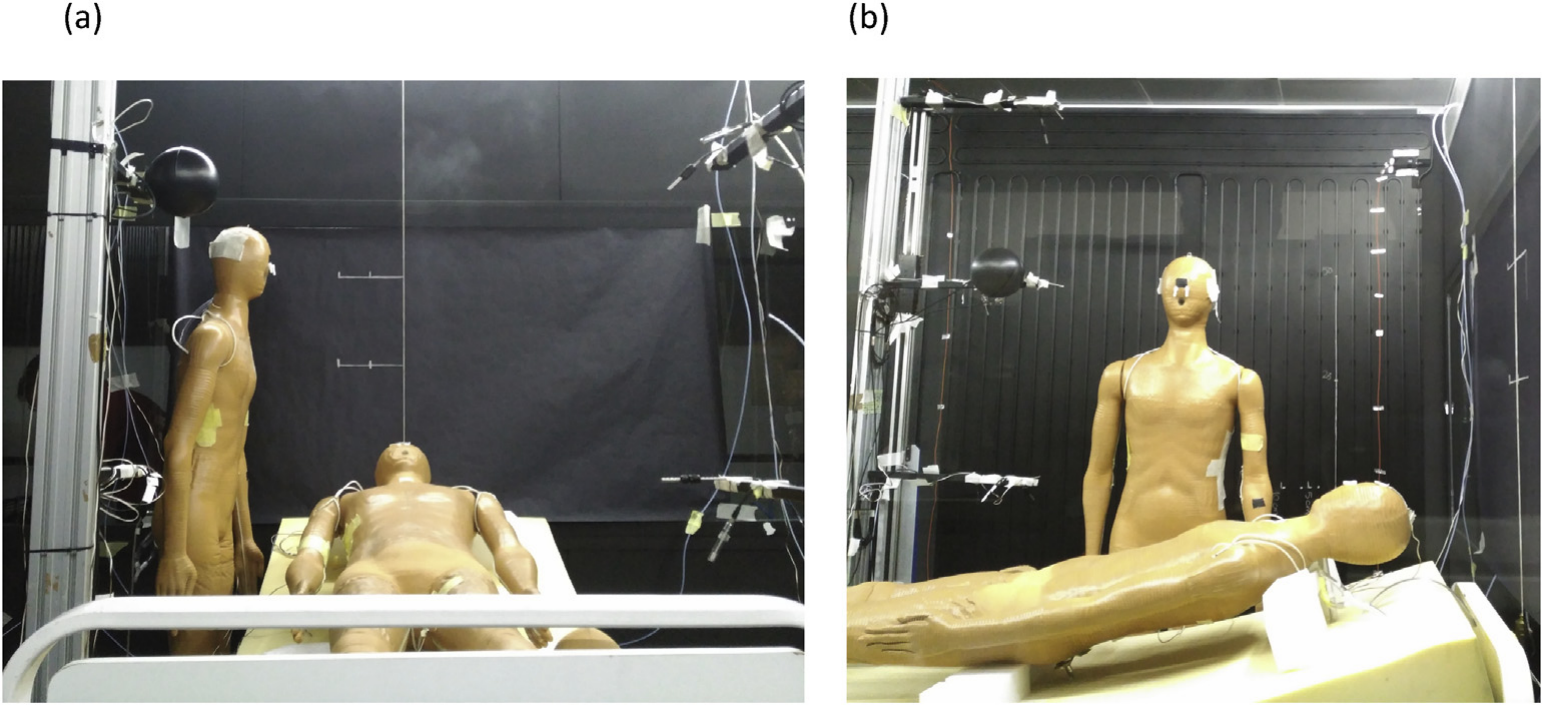
Picture of the experimental room. (a) HW at NM position, (b) HW at NC position with RW at the back.

The number of air changes per hour used in the room have been 6 and 12 h^−1^. External heat gains simulated by a radiant wall (RW) have been simulated operating at 0 W/m^2^ (RW OFF) and 39.7 W/m^2^ (RW ON). The position of HW respect to PR has been modified in order to obtain the influence in the concentration of contaminants inhaled by HW. That corresponds to a total of 16 experiments, see Table 3. For more detailed information about the experimental set up see Ref. [1]. Fig. 2 shows two pictures of the manikins with HW placed at different positions.

The measurements were started after reaching steady state conditions of the experimental room, which were usually achieved after about 10 hours of operation of all the systems. For each case two type of tests were made, test 1 and test 2. Test 1 was carried out in order to obtain information about temperature and contaminant distribution in the microenvironment of HW. For this test five channels of INNOVA device were used, three in P1 and the two extra ones in both exhausts. Pole P1 is moved together with HW, keeping their relative separation distance, for the different positions studied (NC, NM, OC and OM). Test 2 is carried out in order to measure the exposure of HW to the contaminants exhaled by PR. For that test, only one tube of the INNOVA device was used, connected to the inhalation tube of HW. This strategy of measurements makes it possible to obtain more measurement data of the contaminant concentration that reaches HW inhalation over 2 hours, specifically 204 samples of inhaled contaminants were analyzed. Test 2 was carried out immediately after test 1 for the 16 experiments and without entering the room. The values of the contaminant concentration in the exhausts measured in tests 1 are used to obtain the normalized exposure of HW in test 2 since the setup of experiments was the same and the concentration in the exhaust is stable over time. In order to evaluate the stability of the transient measurements in the two exhausts the standard deviation is calculated as data dispersion index as follows:

(1) $$S_{x}=\sqrt{\frac{\sum_{i=1}^{n}{\left(c_{i}-\bar{c}\right)}^{2}}{n-1}}$$

being n the number of data considered, $c_{i}$ each of concentration values registered in the exhaust and $\bar{c}$ the average concentration in the exhaust during the experiment.

**Fig. 3:**
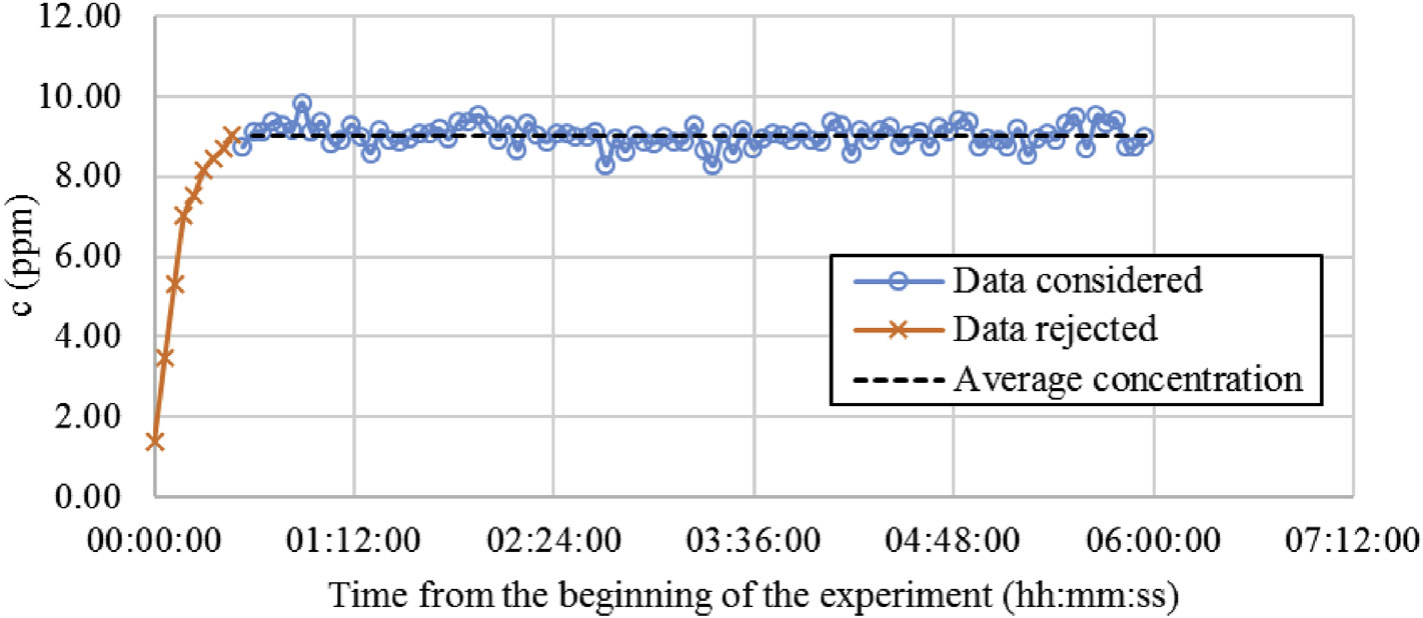
Representation of the exhaust tracer gas concentration in O grille, RW is active and the air ventilation rate considered is 6 ACH.

The values obtained for all the experimental tests considering each of the exhaust routes, the grille near the radiant wall (N) and the placed opposite the radiant wall (O) can be seen in Table 4. An example of the measurements derive over time is shown in Fig. 3.

**Table 4:** Standard deviation of the tracer gas measurements in the ventilation exhausts.

Air Ventilation rate	6 ACH				12 ACH			
Radiant Panel state	RW ON		RW OFF		RW ON		RW OFF	
Exhaust Grille considered	N	O	N	O	N	O	N	O
*Sx*	1.27	0.27	0.79	0.52	0.13	0.10	0.14	0.13

According to the results shown in Fig. 3, the concentration in the exhaust remains stable once the stabilization time ends. Hence, only the data collected after this time is considered.
